# Hyperthermia: A Potential Game-Changer in the Management of Cancers in Low-Middle-Income Group Countries

**DOI:** 10.3390/cancers14020315

**Published:** 2022-01-09

**Authors:** Niloy R. Datta, Bharati M. Jain, Zatin Mathi, Sneha Datta, Satyendra Johari, Ashok R. Singh, Pallavi Kalbande, Pournima Kale, Vitaladevuni Shivkumar, Stephan Bodis

**Affiliations:** 1 Department of Radiotherapy, Mahatma Gandhi Institute of Medical Sciences, Sevagram, Wardha 442012, India; bharati@mgims.ac.in (B.M.J.); zatinmathi@gmail.com (Z.M.); ashoksingh@mgims.ac.in (A.R.S.); pallavikalbande@mgims.ac.in (P.K.); pournimakale@mgims.ac.in (P.K.); 2 Animal Production and Health Laboratory, Joint FAO/IAEA Division of Nuclear Techniques in Food and Agriculture, Department of Nuclear Sciences and Applications, International Atomic Energy Agency (IAEA), P.O. Box 100, 1400 Vienna, Austria; S.Datta@iaea.org; 3 Johari Digital Healthcare Limited, Jodhpur 342012, India; sjohari@joharidigital.com; 4 Department of Pathology, Mahatma Gandhi Institute of Medical Sciences, Sevagram, Wardha 442012, India; shivkumar@mgims.ac.in; 5 Foundation for Research on Information Technologies in Society (IT’IS), 8004 Zurich, Switzerland; s.bodis@bluewin.ch; 6 Department of Radiation Oncology, University Hospital Zurich, 8091 Zurich, Switzerland

**Keywords:** low-middle-income group countries, cancer, hyperthermia, radiotherapy, chemotherapy, recurrent breast cancers, cervical cancer, head and neck cancers, cost-effective, meta-analysis

## Abstract

**Simple Summary:**

As per the Global Cancer Observatory, in 2020, 59% of all cancers globally have been reported from the low-middle-income group countries (LMICs). Cancers of the breast, cervix and head and neck constitute around one-third of the cancers in the LMICs. Most of them are in advanced stages and thus deemed inoperable. Chemoradiotherapy is usually advocated for treatment of these cases with limited success. Moderate hyperthermia at 40–44 °C is a multifaceted therapeutic modality. It is a potent radiosensitizer, chemosensitizer and enforces immunomodulation akin to “in situ tumour vaccination”. The safety and benefit of addition of hyperthermia to radiotherapy and/or chemotherapy in these sites have been well documented in various phase III randomized clinical trials and meta-analysis. Thus, including hyperthermia in the therapeutic armamentarium of clinical care, especially in the LMICs could be a potential game-changer and provide a cost-effective addendum to the existing therapeutic options, especially for these tumour sites.

**Abstract:**

Loco-regional hyperthermia at 40–44 °C is a multifaceted therapeutic modality with the distinct triple advantage of being a potent radiosensitizer, a chemosensitizer and an immunomodulator. Risk difference estimates from pairwise meta-analysis have shown that the local tumour control could be improved by 22.3% (*p* < 0.001), 22.1% (*p* < 0.001) and 25.5% (*p* < 0.001) in recurrent breast cancers, locally advanced cervix cancer (LACC) and locally advanced head and neck cancers, respectively by adding hyperthermia to radiotherapy over radiotherapy alone. Furthermore, thermochemoradiotherapy in LACC have shown to reduce the local failure rates by 10.1% (*p* = 0.03) and decrease deaths by 5.6% (95% CI: 0.6–11.8%) over chemoradiotherapy alone. As around one-third of the cancer cases in low-middle-income group countries belong to breast, cervix and head and neck regions, hyperthermia could be a potential game-changer and expected to augment the clinical outcomes of these patients in conjunction with radiotherapy and/or chemotherapy. Further, hyperthermia could also be a cost-effective therapeutic modality as the capital costs for setting up a hyperthermia facility is relatively low. Thus, the positive outcomes evident from various phase III randomized trials and meta-analysis with thermoradiotherapy or thermochemoradiotherapy justifies the integration of hyperthermia in the therapeutic armamentarium of clinical management of cancer, especially in low-middle-income group countries.

## 1. Introduction

### 1.1. Cancer Status in Low-Middle-Income Group Countries

**Figure 1:**
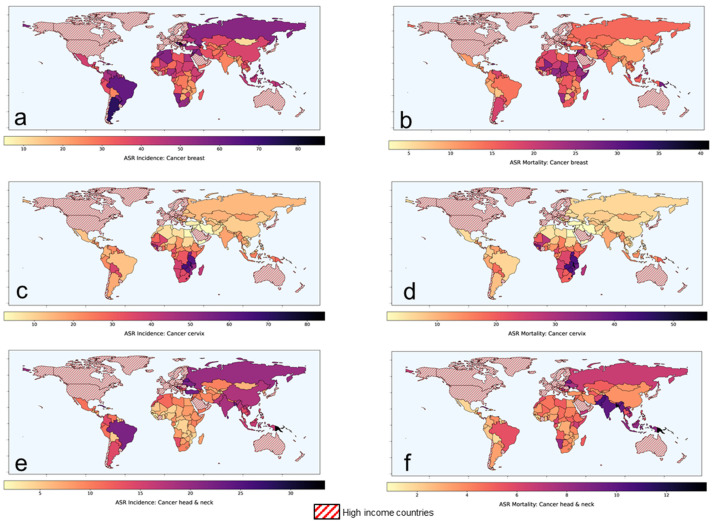
Age-standardized rates of (ASR) for incidence and mortality for (**a**,**b**) breast cancer (**c**,**d**) cervical cancer and (**e**,**f**) head and neck cancers, respectively in low-middle-income group countries. Based on data from Global Cancer Observatory [1].

According to the Global Cancer Observatory of the World Health Organization (WHO), the total cancer incidence estimated in 2020 was 19.3 M and is expected to rise to 24.1 M in 2030 [1]. In 2020, 11.4 M (59%) of these cases were reported in the low-middle-income countries (LMICs) where the cancer burden is projected to escalate to 14.6 M (+28.2%) and 17.9 M (+56.8%) by 2030 and 2040, respectively. Of the 11.4 M cancer cases in LMICs, presently, cancers of breast, cervix and head and neck regions combined constitutes around 3 M (26.2%) of cases. Furthermore, the cancers in these sites in LMICs constitute 61.1%, 88.1% and 71.8% of the global cancers, respectively (Table 1). In view of the advanced stages of their presentation, most of these cases are inoperable. Thus, radiotherapy (RT) and/or chemotherapy (CT) forms the mainstay of their treatment, resulting in a %mortality/incidence at 36%, 58.7% and 38.2% in cancers of the breast, cervix and head and neck, respectively in LMICs (Figure 1, Table 1). Certainly, there is a need to explore other cost-effective options to improve these treatment outcomes in LMICs [2].

### 1.2. Hyperthermia as a “Potential Game-Changer”

Loco-regional hyperthermia (HT) or thermotherapy, at 40–44 °C, has been shown to be a potent radiosensitizer, a chemosensitizer and an immunomodulator with no significantly added side effects [3,4,5]. HT sensitizes the hypoxic tumour cells and inhibits the repair of RT- and/or CT-induced DNA damage. In addition, cells in radioresistant “S” phase are heat sensitive [3]. Furthermore, thermoradiobiologically, HT has been shown to impart high LET properties to low LET proton or photon beams [6]. The addition of HT to photons creates a radiobiological advantage in tumours akin to fast beam neutrons. The physiological vasodilation at temperatures of 39–45 °C allows rapid heat dissipation from normal tissues, thereby sparing the normal tissues from HT-induced morbidity. On the contrary, the chaotic and relatively rigid tumour vasculature results in heat retention leading to higher intratumoural temperatures. Consequently, the high LET attributes of HT with photon radiations are mostly limited to the confines of the heated tumour, while the normothermic normal tissues get irradiated with low LET photons. HT thereby augments photon therapy by conferring therapeutic advantages of high LET radiations to the tumours akin to neutrons, while the ‘heat-sink’ effect spares the normal tissues from thermal radiosensitization. Thus, photon thermoradiotherapy imparts radiobiological advantages selectively to tumours analogous to neutrons without exaggerating normal tissue morbidities.

Accordingly, HT could be an effective therapeutic modality in conjunction with RT and/or CT. Moderate HT, as defined by the Kadota Forum in 2008, is elevation of the tumour temperature between 39 °C and 45 °C [7]. The biological and physiological mechanisms involved in HT at 38–45 °C has been very aptly summarized by van Rhoon [8]. The thermodynamic changes are initiated at around 38 °C and results in a gradual increase in tumour blood flow and subsequent oxygenation while the thermoradiobiological mechanisms lead to direct cell kill, thermal sensitization and inhibition of DNA repair between 39 °C and 45 °C. Thus, at the usual clinically achievable temperature of 40–42 °C, HT could lead to appreciable radiosensitization, chemosensitization and immunomodulation along with RT ± CT.

Incorporating HT along with the standard therapeutic modalities, namely RT and/or CT, could thus be expected to augment therapeutic outcomes through the multifaceted actions of HT [3,4]. In LMICs, most patients present in relatively locally advanced stages, thereby limiting the role of primary surgical option. Thus, RT and/or CT forms the mainstay of management of these locally advanced tumours—namely of head and neck, cervix and breast. The treatment needs to be tolerable as patients usually have compromised nutritional status, especially in LMICs. In addition, due to limited health insurance coverage, most patients may have to bear the cost of their treatment through out-of-pocket resources. All these factors, enforces one to consider cost-effective strategies that are also tolerable with low acute and late morbidities. HT, a safe modality, with limited toxicities, and a known potentiator of RT and CT could thus be a possible therapeutic addendum in clinical settings in LMICs. The present report summarizes the available clinical evidence to justify the inclusion of HT in the management of these common cancers in LMICs along with RT ± CT. As evident, HT could indeed emerge as a potential game-changer by improving the therapeutic outcome in the common cancers prevalent in LMICs.

## 2. Locally Advanced Breast Cancers: Scope for Improvement with Hyperthermia

Locally advanced breast cancers (LABC) are a fairly common problem in LMICs. Most patients present in an advanced stage where primary surgical intervention is usually not feasible. Thus, patients are usually subjected to neoadjuvant chemotherapy (NACT) to enable tumour downstaging followed by mastectomy. Most CT drugs exhibit thermal synergism by (a) increasing the cellular uptake of drugs, (b) increased oxygen radical production, (c) increasing DNA damage, and (d) inhibiting chemotherapeutic-induced DNA damage [9,10,11]. HT inflicts oxidative damage and/or strand cross links, as well as single or double strand DNA breaks, along with CT agents, namely adriamycin, cyclophosphamide, 5-flurouracil and taxanes commonly used as NACT agents for LABC. Further, HT also interferes with the various DNA repair process involving excision repair, non-homologous end joining and/or homologous recombination [9,11].

### Clinical Outcomes with Hyperthermia in Locally Advanced Breast Cancers

In a recently reported randomized clinical trial in stages IIB-IIIA breast cancers, patients treated with NACT (adriamycin, cyclophosphamide, 5-flurouracil) with loco-regional HT using 27.1 MHz, experienced a significant reduction in both primary tumour (+15.9%, *p* = 0.034) and axillary lymph nodes (+14.1%, *p* = 0.011) compared to those treated with NACT alone [12]. Further, a higher proportion of patients underwent breast conservative surgery (+13.6%) with NACT + HT following appreciable tumour regression. A significantly improved overall survival at 10-year was also evident in patients treated with NACT + HT (*p* = 0.009).

In a phase I/II study, Vujaskovic et al. [13], evaluated the safety and efficacy of a NACT with paclitaxel, liposomal doxorubicin and HT in LABC. A combined response rate of 72% was reported at the end of NACT with four of the 43 patients achieving a complete response (CR). A 4-year disease-free and overall survival rate of 63% and 75% were attained, respectively.

HT has been reported to increase the systolic blood flow in breast tumours by about 3.5 times compared to pre-HT blood flow [12].

Thus, NACT + HT could be a viable option for LABC and the consequence of its effects on the key outcomes need to be examined systematically in future studies. These should also incorporate a detailed histopathological evaluation to explore HT-induced immunomodulation.

## 3. Recurrent Breast Cancers and Other Cancers: Scope for Improvement with Hyperthermia

Locoregional recurrence in breast cancers has been reported in one-third of the patients with 80% of these recurrences evident within the first 5 years of primary treatment [14]. Although surgery is the preferred initial option, its role is restricted mostly to operable lesions. The efficacy of CT is yet to be established as evident from a Cochrane review [15]. In an open label randomized study, the efficacy of chemotherapy was limited only to resected oestrogen receptor negative local recurrences [16]. RT alone has been tried in several studies. However, in patients with previously irradiated chest wall, reirradiation (ReRT) with high ReRT doses could lead to a higher risk of radiation-induced normal tissue morbidity depending on the organs at risk, previous dose of irradiation, total RT dose, dose/fraction and the time interval between the first and proposed ReRT.

### Clinical Outcomes with Hyperthermia in Recurrent Breast Cancers

HT, being a potent radiosensitizer, has been used in various clinical studies along with RT as thermoradiotherapy (HTRT) [14]. These include both phase II single arm and phase III randomized control studies. Variable RT doses (24–60 Gy), dose/fraction (1.8–4 Gy/fr) have been explored. HT has been delivered with either microwaves or radiofrequencies (8–2450 MHz) as one to two weekly sessions (total number of sessions, mean: 6.3 ± 2.7) with variable sequences of HT and RT (both before and after RT), based on the institutional protocols and availability of HT equipment. An average temperature of 42.5 °C was attained with HT of 30–90 min duration.

Oldenberg et al. [17] recently reported the efficacy of ReRT with HT in 196 patients of unresectable locoregional recurrent breast cancer en cuirasse who had received a prior RT of 50 Gy. ReRT was delivered as 8 fractions of 4 Gy each or 12 fractions of 3 Gy each along with locoregional HT once or twice a week. An overall clinical response of 72% with a CR of 30% was reported.

**Figure 2:**
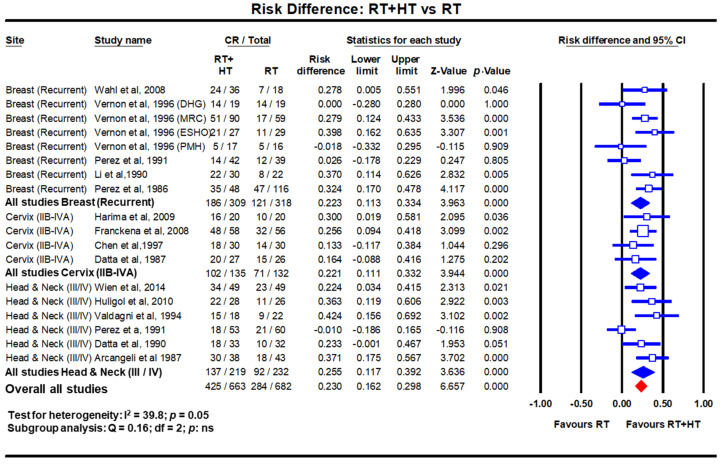
Forest plots depicting the risk difference for complete response with radiotherapy (RT) with hyperthermia (HT) versus RT alone in recurrent breast cancers, locally advanced cervical cancer (stages IIB-IVA) and locally advanced head and neck cancers (stages III/IV). Data extracted from Datta et al. [14,18,19] and replotted. Addition of hyperthermia to radiotherapy favours the outcome compared to radiotherapy alone in all sites with a risk difference of 23% (*p* < 0.001). (Q test: test for heterogeneity; df: degree of freedom and ns: not significant). For citations of the studies listed, please refer to [14,18,19].

A meta-analysis evaluated the efficacy of HTRT over RT alone in recurrent breast cancers [14]. This included 34 studies of which eight were 2-arm comparative trials (*n* = 627 patients) while 26 pertained to single arm studies (*n* = 1483 patients). In the 2-arm studies, a CR of 60.2% vs. 38.1% was evident with HTRT vs. RT alone (odds ratio: 2.64, *p* < 0.001). The risk difference in favour of HTRT was 0.22 (*p* < 0.001) (Figure 2). In the 26 single arm studies, 63.4% attained CR with HTRT. Further, even in 779 patients who had been previously irradiated, a 66.6% CR was documented with a mean ReRT dose of 36.7 Gy (SD: ±7.7 Gy). Mean acute and late grade III/IV toxicities were reported as 14.4% and 5.2%, respectively.

Thus, based on the randomized studies and meta-analysis, HT along with RT appears to be an effective and safe palliative modality in recurrent breast cancers. One could expect a CR with HTRT in nearly two-third of the patients. This is around 22% higher than that with RT alone.

In line with the evidence of role of HT along with RT in recurrent breast cancers, this could be also extended to recurrent tumours of head and neck, cervix and other sites, especially those which have been preirradiated. As seen in recurrent breast tumours, a moderate dose of RT along with a few fractions of HT could be systematically investigated for recurrent tumours in other sites.

## 4. Locally Advanced Cervical Cancer: Scope for Improvement with Hyperthermia

Of all the cervical cancer reported globally in 2020, LMICs account for 88.1% of all cases and 91.4% of all mortalities [1] (Figure 1, Table 1). Thus, the %mortality/incidence in LMICs is estimated at 58.7%. This could be attributed to presentation in most patients in LMICs as locally advanced cervical cancer (LACC). Following the National Cancer Institute guidelines in 1992 [20], chemoradiotherapy (CTRT) using cisplatin as single or in combination is the most common therapeutic intervention in LACC. In a meta-analysis from 14 randomized clinical trials which included 2445 patients, CTRT has been shown to improve the CR (+10.2%, *p* = 0.027), locoregional control (+8.4%, *p* < 0.001) and overall survival (+7.5%, *p* < 0.001) over RT alone [21]. Thus, even though CTRT has shown to improve outcomes over RT alone, it appears that there could still be scope to explore for a possible improvement.

### Clinical Outcomes with Hyperthermia in Locally Advanced Cervical Cancer

**Figure 3:**
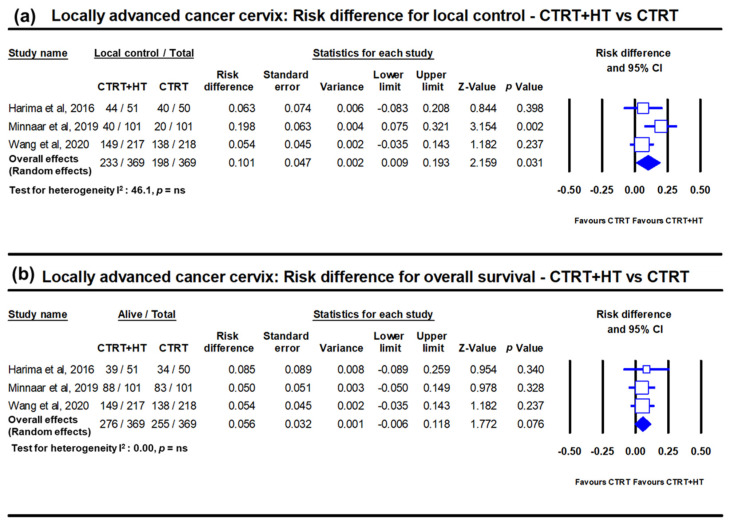
Forest plots depicting the risk difference in locally advanced cancer cervix for (**a**) local disease control and (**b**) overall survival with chemoradiotherapy (CTRT) with hyperthermia (HT) versus CTRT alone. Data from Minnaar et al. [23] has been added to the meta-analysis from Yea et al. [22] and replotted. The risk difference for local failure with HT added to CTRT reduces by 10.1% (*p* = 0.03) while the overall survival improves by 5.6% (*p* = 0.07). (ns: not significant). For citations of the studies listed, please refer to [22,23].

HT has also been used along with RT in several randomized clinical trials in LACC. The outcomes as evident on meta-analysis between HTRT vs. RT, shows a distinct improvement with HTRT in terms of CR at the end of treatment and loco-regional control of 22% (*p* < 0.001) and 23% (*p* < 0.001), respectively (Figure 3) [18]. A non-significant survival advantage of 8.4% with HTRT was also noted without any significant escalation of acute or late morbidities with HT added to RT. Even when HT was used with CTRT, the risk difference from three randomized clinical trials (total patients = 738) for local control and overall survival showed an advantage with HTCTRT over CTRT by 10.1% (*p* = 0.03) and 5.6% (*p*: ns), respectively [22,23] (Figure 3).

**Figure 4:**
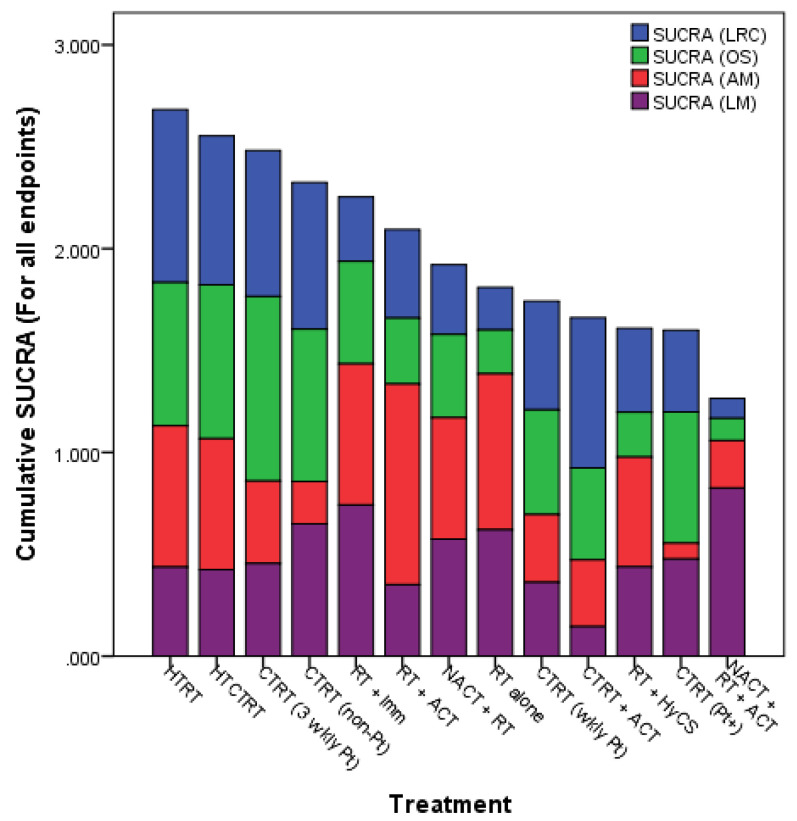
Surface under the cumulative ranking curve (SUCRA) values for endpoints from all studies (1974–2018) in locally advanced cancer cervix. LRC = loco-regional control; OS = overall survival; AM = acute morbidity (grade ≥ 3); LM = late morbidity (grade ≥ 3) (Reproduced with permission from Datta et al. [24]).

Network meta-analysis, which provides the highest level of clinical evidence, was reported in LACC, in which all the 13 different therapeutic approaches were evaluated from 49 clinical trials totalling 9894 patients [24]. The surface under cumulative ranking curve (SUCRA) estimates provide an objective assessment and ranking of the locoregional control, overall survival, acute and late morbidity. The SUCRA values ranked all the 13 different strategies used in randomized clinical trial settings. Incidentally, the top two approaches evident on SUCRA values were HTRT and HTCTRT in LACC (Figure 4).

Thus, based on the highest levels of clinical evidence obtained through both conventional pairwise and network meta-analysis, HT with either RT or CTRT appears to provide a superior therapeutic benefit even when compared to the standard practice of CTRT in LACC. Moreover, HT has been shown to be safe with no significant additional acute or late morbidity to RT or CTRT. It would therefore be pertinent to incorporate HT in the routine clinical management of LACC along with RT or CTRT. This may help to mitigate the high %mortality/incidence seen in cervical cancer in LMICs.

## 5. Locally Advanced Head and Neck Cancers: Scope of Improvement with Hyperthermia

In 2020, 71.8% and 81.5% of all global incidence and mortalities in head and neck cancers were reported in the LMICs [1]. The %mortality/incidence in LMICs for these cancers are estimated at 38.2% (Figure 1, Table 1). As in the cervix, most patients present as locally advanced head and neck cancers (LAHNC), CTRT has been the mainstay of their treatment. CTRT has been shown to improve outcomes in successive reports of the Meta-analysis of Chemotherapy in Head and Neck (MACH-NC) collaborative group. In their latest update of 107 randomized trials with 19,085 patients published in 2021, a 6.5% absolute benefit at 5 years was demonstrated (hazard ratio: 0.83; 95% CI: 0.79–0.86) [25]. However, this benefit reduced with increasing patient age and poor performance status.

### Clinical Outcomes with Hyperthermia in Locally Advanced Head and Neck Cancer

In LMICs, patients with LAHNC are often in poor performance status due to inadequate nutritional intake. This could have a bearing on the outcomes with CTRT. HT has been used with RT and outcomes compared with RT alone. In a meta-analysis of six clinical trials comprising 451 cases of LAHNC, HTRT improved the overall CR by 25.5% over RT alone (*p* < 0.0001) [19] (Figure 2). Acute and late morbidities appear similar.

The positive outcomes of HTCTRT in LACC, which also share a similar histology with LAHNC, should encourage patients to be recruited for phase III randomized trial with HTCTRT vs. CTRT alone. However, one of limitations could be lack of a proper HT unit for head and neck region that would allow adequate heating and monitoring of HT during individual treatment session. A dedicated HT delivery system working at 433 MHz–the HYPERCollar (Sensius, Rotterdam, The Netherlands) fills in the long-standing gap for a site-specific HT for LAHNC [26,27,28,29,30,31,32]. The system initially had 12 antennas, which was later upgraded to 20 antennas. Presently, an MR-compatible version of this applicator is being used within a 1.5 T MR system. This would allow online monitoring of the temperature using non-invasive thermometry with the proton resonance frequency shift method [33,34]. In addition, model-based and other new MR-thermometry temperature reconstruction methods are emerging which are quite promising [35,36,37]. The unit is currently being validated in clinics for HT delivery in head and neck regions [38].

Thus, LAHNC provides yet another common site in which HT, along with RT or CTRT, could be expected to improve therapeutic outcomes without any significant added toxicities. It is therefore highly desirable that HT should be evaluated systematically in LAHNC. As LMICs harbour more than two-thirds of global head and neck cancers, these patients need to be included in single/multicentric clinical trials for evaluating HTCTRT vs. CTRT alone.

## 6. Setting Up a Hyperthermia Treatment Facility in Low-Middle-Income Countries

### 6.1. Choice of Hyperthermia Unit for LMICs

Local HT treatments could be delivered by a host of methods—external HT (radiative or capacitive), local invasive (intraluminal and interstitial), regional perfusion or water-filtered infra-red [7,39]. Clinical HT is usually delivered using radiofrequency (radiative or capacitive), microwaves (434–915 MHz), ultrasound or infrared (>300 GHz) devices. A detailed technical description of each of these methods is beyond the scope of this manuscript. Readers may like to refer to the European Society for Hyperthermia Oncology (ESHO) guidelines that also gives a detailed descriptions of these devices for use in clinics [40,41,42,43]. However, due to different heating patterns in depth, the choice of equipment, especially in a resource constraint situation, should preferably be based on the type of common tumours prevalent in the geographical area to be catered by the institution. Even the instrument design and the choice of frequencies of the radiative or capitative systems would need to be selected based on the tumour site and its depth that would be commonly treated by a centre [44]. In addition, the availability of trained personnel for HT treatment delivery, thermometry systems for online temperature monitoring, and the resources allocated, need to be considered before planning to set up such a facility. Presently, HT is not available in most LMICs, and therefore, all these factors would have to be carefully weighed before one launches into such a programme.

Radiofrequency capacitive systems operating at 27.1 MHz are cheap and are commonly used in most of the physiotherapy centres in LMICs as a short-wave diathermy. These units are based on plane-wave matching, in which the antenna’s plane-parallel plates are tuned as per the standard antenna-tuning method [45]. The target tissue placed between the condenser plates act as a capacitor to store electrical charge, resulting in local heating of the tissue. Heat is induced by the resulting currents and is directed toward the smallest electrode [44,46]. Capacitive heating creates high power densities around the bolus edges, but one needs to be careful due to its preferential heating in the subcutaneous fat layer. This may be of special relevance to obese patients with considerable subcutaneous fat. Thus, these units need a circulatory water bolus to have adequate skin cooling. The operation of the unit is relatively simple and technical staff can be easily trained on these units compared to the other state-of-the-art commercially available HT units that are based on radiative/microwave technologies, some of which could also be compatible with MRI thermometry. However, fibreoptic single or multi-sensor radiofrequency immune thermometry probes or thermocouples are required for continuous temperature monitoring. Additional components for thermal simulation and treatment planning supported by quality assurance needs to be introduced for a better temperature assessment in the heated volumes. It should be reasonably feasible to treat the common tumour sites in LMICs–LAHNC, LABC and LACC using 27.1 MHz, after incorporating a circulatory water bolus for surface skin cooling and thermometry for temperature monitoring. However, the 27.1 MHz capacitive heating device would not allow non-invasive thermometry as feasible with some of the MR-compatible versions of HT delivery currently available commercially (HYPERCollar from Sensius and BSD-2000 3D/MR from Pyrexar Medical, Salt Lake City, UT, USA).

Capacitive heating using 27.1 MHz radiofrequency has been used clinically for HT in some clinical studies [12,47] with satisfactory outcomes. As discussed earlier, the recently reported randomized phase III trial NACT + HT vs. HT in stages IIA-IIIB was conducted using 27.1 MHz in 200 patients [12]. This had resulted in a significant favourable outcome for patients treated with NACT + HT in terms of the objective response rate, the proportion of women eligible for breast-conserving and reconstructive surgery and the 10-year overall survival rates compared with NACT alone. The objective response at the primary site was reported to be higher by 15.9% with HT + NACT compared to NACT alone (*p* = 0.034). Correspondingly in the lymph nodes, the response was higher by 14.1% (*p* = 0.011). Computer-assisted planning helped to select a personalized distribution of the magnetic, electric and thermal fields generated by the unit.

### 6.2. Cost Computations and Its Implications for a Hyperthermia Setup in LMICs

HT units cost a fraction of the RT units and is a one-time investment with minimal recurring costs. These usually have a working life of 10 years. Unlike RT, the daily patient throughput is lower as each treatment may take around 90 min. In an 8 h working day, four to five patients can be treated/day/unit, that is, 20–25 patients/week, as HT is usually delivered once or twice a week. Thus, 170–325 patients/year could be treated with HT, if delivered once a week for 4–6 weeks. This may go down to 85–162 patients, if twice a week HT treatment scheduled is adopted by a department [3].

Thus, a centre may need to compute the break-even point (BEP) and % return on investment (%ROI) following capital investment to set up a HT facility. Assuming, the capital cost to set up a HT unit is “C” USD, number of patients treated with HT per year as “N” and user cost as “U” USD, the BEP would be

(1) $$\mathrm{BEP}=\frac{C}{\mathrm{NU}}\;\left(\mathrm{in}\;\mathrm{years}\right).$$

Assuming that the HT unit has a working life of 10 years, the income generated in the post-BEP period would be estimated as,

(2) $$\mathrm{Income}\;\mathrm{generated}\;\mathrm{in}\;\mathrm{the}\;\mathrm{post}-\mathrm{BEP}\;\mathrm{period}\;=\left(10-\frac{C}{\mathrm{NU}}\right)\times \;\mathrm{NU}.$$

Thus,

(3) $$\%\;\mathrm{ROI}\;=\frac{\mathrm{Income}\;\mathrm{generated}\;\mathrm{in}\;\mathrm{the}\;\mathrm{post}-\mathrm{BEP}\;\mathrm{period}\;-\;\mathrm{Cos}t\;\mathrm{of}\;\mathrm{investment}\;}{\mathrm{Cos}t\;\mathrm{of}\;\mathrm{investment}}\times 100=\frac{10\mathrm{NU}-2C}{C}\times 100.$$

Using the above expressions, any department in any country can work out the optimal BEP and %ROI based on the planned capital investment, number of patients estimated to be treated per year and the user cost. The investment for HT unit could vary and depend on the availability of resources–both financial and human. The corresponding returns would hinge on the patient load, treatment charges, working schedule and departmental policy of weekly or biweekly HT treatments. Cost computations and the %returns on investment (%ROI) need to be computed by individual countries, taking into consideration the above factors, as this may vary from country to country. A cost of EUR 6800 was computed for a series of five treatments in the Dutch Deep Hyperthermia Trial, of which half of the amount was for personnel and one-third for equipment [7]. This is likely to be much lower in LMICs and hence higher returns could be expected. HT could contribute to just a minimal fraction of the cost to the primary treatment in comparison to most standard CT regimes and also immunotherapies, which are being increasingly advocated in many tumour sites. This would not only help to bring down the treatment cost, but also make it more affordable, tolerable and by virtue of improving the therapeutic outcomes, could also improve the quality of life with least morbidity.

## 7. Conclusions

Apart from being a cost-effective option, HT provides several tangible and nontangible gains and should be explored in LMICs. The tangible gains would comprise cost of treatment, cost efficacy, response rates, survival, etc. while the nontangible would be more subjective and include wellbeing of the patients, reporting back to work early, supporting their families, etc. It is perhaps time to integrate HT in the therapeutic management of cancers, especially the locally advanced and recurrent tumours as seen in LMICs. Though the efficacy of HT has been discussed in three specific sites of LAHNC, LACC, LABC and recurrent breast cancers which are common in LMICs, the benefit of HT with RT ± CT has been documented in various sites, namely superficial tumours, melanoma, choroidal melanoma, brain tumours, malignant germ cell tumours, soft tissue sarcoma, bone metastases, oesophagus, lung, pancreas, urinary bladder, prostate, rectum, anus and others [3,4,48,49,50]. Clinical evidence indicates a steady benefit of integrating HT with the standard treatments in most sites.

Thus, based on the above thermoradiobiological rationale and clinical evidence, HT could certainly prove to be a “potential game-changer” when integrated in the therapeutic strategies for various malignancies, especially those with locally advanced tumours as prevalent in LMICs. HT is a cost-effective and a unique multifaceted treatment modality and deserves to be incorporated in the present-day clinical oncology practice and management.

## Figures and Tables

**Table 1:** Estimated number of cancer cases and deaths as per the Global Cancer Observatory in ages (0–85+ years) pertaining to breast, cervix and head and neck region globally and in low-middle-income countries (LMICs) in 2020 [1]. Countries classified in various income groups based on the World Bank classification.

Cancer Sites	Cancer Incidence			Cancer Mortality			% Mortality/Incidence in LMICs
	All Countries	LMICs Only	Proportion in LMICs (%)	All Countries	LMICs Only	Proportion in LMICs (%)	
All sites	19,292,789	11,441,886	59.3	9,958,133	7,063,070	70.9	61.7
Breast	2,261,419	1,381,539	61.1	684,996	497,496	72.6	36.0
Cervix	604,127	532,239	88.1	341,831	312,373	91.4	58.7
Head and neck ^#^	1,518,133	1,090,262	71.8	510,771	416,206	81.5	38.2

^#^ includes cancers of lip, oral cavity, nasopharynx, oropharynx, hypopharynx, larynx, salivary glands and thyroid; Data as on 12 September 2021 [1].

## References

[1] Ferlay J., Ervik M., Lam F., Colombet M., Mery L., Piñeros M., Znaor A., Soerjomataram I., Bray F. (2020). Global cancer observatory: Cancer Today. Book Global Cancer Observatory: Cancer Today.

[2] Sankaranarayanan R., Swaminathan R., Jayant K., Brenner H., Sankaranarayanan R., Swaminathan R., Lucas E. (2011). An overview of cancer survival in Africa, Asia, Caribbean and Central America. Cancer Survival in Africa, Asia, the Caribbean and Central America.

[3] Datta N.R., Kok H.P., Crezee H., Gaipl U.S., Bodis S. (2020). Integrating loco-regional hyperthermia into the current oncology practice: SWOT and TOWS analyses. Front. Oncol..

[4] Datta N.R., Ordonez S.G., Gaipl U.S., Paulides M.M., Crezee H., Gellermann J., Marder D., Puric E., Bodis S. (2015). Local hyperthermia combined with radiotherapy and-/or chemotherapy: Recent advances and promises for the future. Cancer Treat. Rev..

[5] Frey B., Weiss E.M., Rubner Y., Wunderlich R., Ott O.J., Sauer R., Fietkau R., Gaipl U.S. (2012). Old and new facts about hyperthermia-induced modulations of the immune system. Int. J. Hyperth..

[6] Datta N.R., Bodis S. (2019). Hyperthermia with photon radiotherapy is thermo-radiobiologically analogous to neutrons for tumours without enhanced normal tissue toxicity. Int. J. Hyperth..

[7] Van der Zee J., Vujaskovic Z., Kondo M., Sugahara T. (2008). The Kadota Fund International Forum 2004--clinical group consensus. Int. J. Hyperth..

[8] Van Rhoon G.C. (2016). Is CEM43 still a relevant thermal dose parameter for hyperthermia treatment monitoring?. Int. J. Hyperth..

[9] Issels R.D. (2008). Hyperthermia adds to chemotherapy. Eur. J. Cancer.

[10] Issels R. (1999). Hyperthermia combined with chemotherapy—Biological rationale, clinical application, and treatment results. Oncol. Res. Treat..

[11] Oei A.L., Vriend L.E., Crezee J., Franken N.A., Krawczyk P.M. (2015). Effects of hyperthermia on DNA repair pathways: One treatment to inhibit them all. Radiat. Oncol..

[12] Loboda A., Smolanka I., Orel V.E., Syvak L., Golovko T., Dosenko I., Lyashenko A., Smolanka I., Dasyukevich O., Tarasenko T. (2020). Efficacy of combination neoadjuvant chemotherapy and regional inductive moderate hyperthermia in the treatment of patients With locally advanced breast cancer. Technol. Cancer Res. Treat..

[13] Vujaskovic Z., Kim D.W., Jones E., Lan L., McCall L., Dewhirst M.W., Craciunescu O., Stauffer P., Liotcheva V., Betof A. (2010). A phase I/II study of neoadjuvant liposomal doxorubicin, paclitaxel, and hyperthermia in locally advanced breast cancer. Int. J. Hyperth..

[14] Datta N.R., Puric E., Klingbiel D., Gomez S., Bodis S. (2016). Hyperthermia and radiation therapy in locoregional recurrent breast cancers: A systematic review and meta-analysis. Int. J. Radiat. Oncol. Biol. Phys..

[15] Rauschecker H., Clarke M., Gatzemeier W., Recht A. (2001). Systemic therapy for treating locoregional recurrence in women with breast cancer. Cochrane Database Syst. Rev..

[16] Wapnir I.L., Price K.N., Anderson S.J., Robidoux A., Martín M., Nortier J.W.R., Paterson A.H.G., Rimawi M.F., Láng I., Baena-Cañada J.M. (2018). Efficacy of chemotherapy for ER-negative and ER-positive isolated locoregional recurrence of breast cancer: Final analysis of the CALOR trial. J. Clin. Oncol..

[17] Oldenborg S., Rasch C.R.N., van Os R., Kusumanto Y.H., Oei B.S., Venselaar J.L., Heymans M.W., Zum Vörde Sive Vörding P.J., Crezee H., van Tienhoven G. (2018). Reirradiation + hyperthermia for recurrent breast cancer en cuirasse. Strahlenther. Onkol..

[18] Datta N.R., Rogers S., Klingbiel D., Gomez S., Puric E., Bodis S. (2016). Hyperthermia and radiotherapy with or without chemotherapy in locally advanced cervical cancer: A systematic review with conventional and network meta-analyses. Int. J. Hyperth..

[19] Datta N.R., Rogers S., Ordonez S.G., Puric E., Bodis S. (2016). Hyperthermia and radiotherapy in the management of head and neck cancers: A systematic review and meta-analysis. Int. J. Hyperth..

[20] National Institute of Health, National Cancer Institute (NCI) NCI Issues Clinical Announcement on Cervical Cancer: Chemotherapy Plus Radiation Improves Survival. Chemotherapy Plus Radiation Improves Survival, 1999. http://www3.scienceblog.com/community/older/archives/B/nih478.html.

[21] Datta N.R., Stutz E., Liu M., Rogers S., Klingbiel D., Siebenhuner A., Singh S., Bodis S. (2017). Concurrent chemoradiotherapy vs. radiotherapy alone in locally advanced cervix cancer: A systematic review and meta-analysis. Gynecol. Oncol..

[22] Yea J.W., Park J.W., Oh S.A., Park J. (2021). Chemoradiotherapy with hyperthermia versus chemoradiotherapy alone in locally advanced cervical cancer: A systematic review and meta-analysis. Int. J. Hyperth..

[23] Minnaar C.A., Kotzen J.A., Ayeni O.A., Naidoo T., Tunmer M., Sharma V., Vangu M.D., Baeyens A. (2019). The effect of modulated electro-hyperthermia on local disease control in HIV-positive and -negative cervical cancer women in South Africa: Early results from a phase III randomised controlled trial. PLoS ONE.

[24] Datta N.R., Stutz E., Gomez S., Bodis S. (2019). Efficacy and safety evaluation of the various therapeutic options in locally advanced cervix cancer: A systematic review and network meta-analysis of randomized clinical trials. Int. J. Radiat. Oncol. Biol. Phys..

[25] Lacas B., Carmel A., Landais C., Wong S.J., Licitra L., Tobias J.S., Burtness B., Ghi M.G., Cohen E.E.W., Grau C. (2021). Meta-analysis of chemotherapy in head and neck cancer (MACH-NC): An update on 107 randomized trials and 19,805 patients, on behalf of MACH-NC Group. Radiother. Oncol..

[26] Paulides M.M., Bakker J.F., Neufeld E., van der Zee J., Jansen P.P., Levendag P.C., van Rhoon G.C. (2007). The HYPERcollar: A novel applicator for hyperthermia in the head and neck. Int. J. Hyperth..

[27] Paulides M.M., Bakker J.F., Zwamborn A.P., Van Rhoon G.C. (2007). A head and neck hyperthermia applicator: Theoretical antenna array design. Int. J. Hyperth..

[28] Paulides M.M., Dobsicek Trefna H., Curto S., Rodrigues D.B. (2020). Recent technological advancements in radiofrequency- andmicrowave-mediated hyperthermia for enhancing drug delivery. Adv. Drug Deliv. Rev..

[29] Paulides M.M., Verduijn G.M., Van Holthe N. (2016). Status quo and directions in deep head and neck hyperthermia. Radiat. Oncol..

[30] Drizdal T., Paulides M.M., van Holthe N., van Rhoon G.C. (2018). Hyperthermia treatment planning guided applicator selection for sub-superficial head and neck tumors heating. Int. J. Hyperth..

[31] Verduijn G.M., de Wee E.M., Rijnen Z., Togni P., Hardillo J.A.U., Ten Hove I., Franckena M., van Rhoon G.C., Paulides M.M. (2018). Deep hyperthermia with the HYPERcollar system combined with irradiation for advanced head and neck carcinoma—A feasibility study. Int. J. Hyperth..

[32] Verhaart R.F., Rijnen Z., Fortunati V., Verduijn G.M., van Walsum T., Veenland J.F., Paulides M.M. (2014). Temperature simulations in hyperthermia treatment planning of the head and neck region: Rigorous optimization of tissue properties. Strahlenther. Onkol..

[33] Gellermann J., Hildebrandt B., Issels R., Ganter H., Wlodarczyk W., Budach V., Felix R., Tunn P.U., Reichardt P., Wust P. (2006). Noninvasive magnetic resonance thermography of soft tissue sarcomas during regional hyperthermia: Correlation with response and direct thermometry. Cancer.

[34] Craciunescu O.I., Stauffer P.R., Soher B.J., Wyatt C.R., Arabe O., Maccarini P., Das S.K., Cheng K.S., Wong T.Z., Jones E.L. (2009). Accuracy of real time noninvasive temperature measurements using magnetic resonance thermal imaging in patients treated for high grade extremity soft tissue sarcomas. Med. Phys..

[35] Poorman M.E., Braskute I., Bartels L.W., Grissom W.A. (2019). Multi-echo MR thermometry using iterative separation of baseline water and fat images. Magn. Reson. Med..

[36] Zhang L., Armstrong T., Li X., Wu H.H. (2019). A variable flip angle golden-angle-ordered 3D stack-of-radial MRI technique for simultaneous proton resonant frequency shift and T1 -based thermometry. Magn. Reson. Med..

[37] Tan J., Mougenot C., Pichardo S., Drake J.M., Waspe A.C. (2019). Motion compensation using principal component analysis and projection onto dipole fields for abdominal magnetic resonance thermometry. Magn. Reson. Med..

[38] Sumser K., Drizdal T., Bellizzi G.G., Hernandez-Tamames J.A., van Rhoon G.C., Paulides M.M. (2021). Experimental validation of the MRcollar: An MR compatible applicator for deep heating in the head and neck region. Cancers.

[39] Notter M., Piazena H., Vaupel P. (2017). Hypofractionated re-irradiation of large-sized recurrent breast cancer with thermography-controlled, contact-free water-filtered infra-red-A hyperthermia: A retrospective study of 73 patients. Int. J. Hyperth..

[40] Trefna H.D., Crezee H., Schmidt M., Marder D., Lamprecht U., Ehmann M., Hartmann J., Nadobny J., Gellermann J., van Holthe N. (2017). Quality assurance guidelines for superficial hyperthermia clinical trials: I. clinical requirements. Int. J. Hyperth..

[41] Trefna H.D., Crezee J., Schmidt M., Marder D., Lamprecht U., Ehmann M., Nadobny J., Hartmann J., Lomax N., Abdel-Rahman S. (2017). Quality assurance guidelines for superficial hyperthermia clinical trials: II. technical requirements for heating devices. Strahlenther. Onkol..

[42] Lagendijk J.J., Van Rhoon G.C., Hornsleth S.N., Wust P., De Leeuw A.C., Schneider C.J., Van Dijk J.D., Van Der Zee J., Van Heek-Romanowski R., Rahman S.A. (1998). ESHO quality assurance guidelines for regional hyperthermia. Int. J. Hyperth..

[43] Bruggmoser G., Bauchowitz S., Canters R., Crezee H., Ehmann M., Gellermann J., Lamprecht U., Lomax N., Messmer M.B., Ott O. (2011). Quality assurance for clinical studies in regional deep hyperthermia. Strahlenther. Onkol..

[44] Kok H.P., Crezee J. (2017). A comparison of the heating characteristics of capacitive and radiative superficial hyperthermia. Int. J. Hyperth..

[45] Szasz A. (2021). The capacitative coupling modalities for oncological hyperthermia. Open J. Biophys..

[46] Abe M., Hiraoka M., Takahashi M., Egawa S., Matsuda C., Onoyama Y., Morita K., Kakehi M., Sugahara T. (1986). Multi-institutional studies on hyperthermia using an 8-MHz radiofrequency capacitive heating device (Thermotron RF-8) in combination with radiation for cancer therapy. Cancer.

[47] Reddy N.M., Maithreyan V., Vasanthan A., Balakrishnan I.S., Bhaskar B.K., Jayaraman R., Shanta V., Krishnamurthi S. (1987). Local RF capacitive hyperthermia: Thermal profiles and tumour response. Int. J. Hyperth..

[48] Datta N.R., Schneider R., Puric E., Ahlhelm F.J., Marder D., Bodis S., Weber D.C. (2016). Proton irradiation with hyperthermia in unresectable soft tissue sarcoma. Int. J. Part. Ther..

[49] Datta N.R., Stutz E., Puric E., Eberle B., Meister A., Marder D., Timm O., Rogers S., Wyler S., Bodis S. (2019). A pilot study of radiotherapy and local hyperthermia in elderly patients with muscle-invasive bladder cancers unfit for definitive surgery or chemoradiotherapy. Front. Oncol..

[50] Rogers S.J., Datta N.R., Puric E., Timm O., Marder D., Khan S., Mamot C., Knuchel J., Siebenhuner A., Pestalozzi B. (2021). The addition of deep hyperthermia to gemcitabine-based chemoradiation may achieve enhanced survival in unresectable locally advanced adenocarcinoma of the pancreas. Clin. Transl. Radiat. Oncol..

